# Intravenous ibuprofen versus ketorolac for perioperative pain control in open abdominal hysterectomy: a randomized controlled trial

**DOI:** 10.1186/s12871-024-02571-0

**Published:** 2024-06-07

**Authors:** Sarah Amin, Ahmed Hasanin, Ola A Attia, Maha Mostafa, Nashwa S Elzayat, Mona Elsherbiny, Amany A Eissa

**Affiliations:** Department of Anesthesia and Critical Care Medicine, Cairo University, Cairo, Egypt

**Keywords:** Ibuprofen, Ketorolac, Open hysterectomy, Postoperative pain, Visual analogue scale

## Abstract

**Background:**

We aimed to compare the analgesic effects of intravenous ibuprofen to ketorolac after open abdominal hysterectomy.

**Methods:**

This randomized double-blinded controlled trial included adult women scheduled for elective open abdominal hysterectomy. Participants were randomized to receive either 30 mg ketorolac (*n* = 50) or 800 mg ibuprofen (*n* = 50) preoperatively, then every 8 h postoperatively for 24 h. All participants received paracetamol 1 gm/6 h. Rescue analgesic was given if the visual analogue scale (VAS) for pain assessment was > 3. The primary outcome was the mean postoperative dynamic VAS during the first 24 h. Secondary outcomes were static VAS, intraoperative fentanyl consumption, postoperative morphine consumption, time to independent movement, and patient’s satisfaction.

**Results:**

Forty-six patients in the ibuprofen group and fifty patients in the ketorolac group were analyzed. The 24-h dynamic and static VAS were similar in the two groups. The median (quartiles) dynamic VAS was 1.1 (0.9, 1.9) in the ibuprofen group versus 1.0 (0.7, 1.3) in the ketorolac group, P-value = 0.116; and the median (quartiles) static VAS was 0.9 (0.6, 1.3) in the ibuprofen group versus 0.7 (0.4, 1.1) in the ketorolac group, P-value = 0.113. The intra- and postoperative analgesic requirements were also similar in the two groups. However, patient satisfaction was slightly higher in the ketorolac group than that in the ibuprofen group (median [quartiles]: 6 [5, 7] versus 5 [4, 7], respectively), P-value: 0.009.

**Conclusion:**

The two drugs, intravenous ibuprofen and ketorolac produced similar analgesic profile in patients undergoing open abdominal hysterectomy receiving multimodal analgesic regimen. NCT05610384, Date of registration: 09/11/2022

**Clinical trial registration:**

ClinicalTrials.gov Identifier: NCT05610384. https://clinicaltrials.gov/ct2/show/NCT05610384

**Supplementary Information:**

The online version contains supplementary material available at 10.1186/s12871-024-02571-0.

## Introduction

Postoperative pain is a major health concern with several adverse outcomes. Postoperative pain after pelvic surgery is associated with patient dissatisfaction, delayed ambulation, prolonged hospital stay, increased rate of readmission and increased risk of chronic postoperative pain [1]. Therefore, proper management of pain after pelvic surgery is one of the important targets for the anesthetist.

The perioperative use of opioids is associated with significant adverse side effects, including nausea, gastrointestinal paralysis, delirium, hypoxemia, hyperalgesia, chronic pain and addiction [2]. Hence, the use of perioperative opioid-sparing strategies, based on regional technique and non-opioids analgesia, had gained increased interest in recent years.

Non-steroidal anti-inflammatory drugs (NSAIDs) are frequently- used drugs in opioid-sparing protocols [3, 4]. Ketorolac and ibuprofen are among the commonest NSAIDs with the advantage of the feasibility of intravenous use. Both drugs had been previously used in various pain management strategies [5, 6]. However, it is unclear which of the two drugs is superior for pain control in surgical patients. Few data evaluated the two drugs in the surgical settings [7, 8]; however, no studies, to the best of our knowledge, compared the two drugs in patients undergoing open laparotomy. Therefore, we conducted this study to compare the use of intravenous ibuprofen to ketorolac for pain control during and after open hysterectomy. We hypothesized that ketorolac would provide superior analgesia to ibuprofen in patients undergoing open abdominal hysterectomy.

## Methods

This randomized double-blinded controlled trial was conducted at a University Hospital between November 2022 and May 2023, after the institutional ethics committee approval (MD-246-2022). Clinical trial registration was done before patients’ enrolment at ClinicalTrials.gov (NCT05610384, Date of registration: 09/11/2022). Informed consent was obtained from the patient before enrolment in the study.

Participants were adult (40–65 years) women with American Society of Anesthesiologist (ASA) classification of I-II, scheduled for open elective abdominal hysterectomy with or without salpingo-oophorectomy.

Exclusion criteria were renal impairment (history of renal impairment or kidney function tests above normal reference range), history of gastrointestinal bleeding or ulceration, inflammatory bowel disease, allergy to any of the study’s drugs, significant cardiac morbidity (impaired contractility, ischemic heart disease). Patients undergoing surgery for suspected gynecological cancer, patients on chronic analgesic medication, and patients deemed unable to understand the visual analogue scale (VAS) were also excluded from the study.

Randomization was done using an online randomizer (https://www.graphpad.com/quickcalcs/randomize1/) in 1:1 ratio. The group assignment and drug preparation instructions were put inside sequentially-numbered opaque envelopes. An independent researcher handled the envelope-opening and drug-preparation. The three scheduled doses were prepared at once; and the unused preparations were stored in the refrigerator and were marked with the patient’s name, hospital number and the time for administration. Ketorolac (Ketolac 30 mg/ 2 mL, AMRIYA PHARM. IND, Alexandria, Egypt) was prepared by diluting 30 mg in 200 mL normal saline; and ibuprofen (Ibuprofen-Arabcomed 100 mg/mL, ARABCOMED, Cairo, Egypt) was prepared by diluting 800 mg in 200 mL normal saline. The attending anesthetist, nurse, data collector and the patient were blinded to the group assignment.

Thirty minutes before surgery, the participants received the first dose of the study drug according to the group assignment in addition to 1 gm intravenous paracetamol (Medalgesic 10 mg/mL, ARABCOMED, Cairo, Egypt). The study drug was then given every 8 h and paracetamol was given every 6 h for 24 h.

In the operating room, electrocardiogram, pulse oximetry, and non-invasive blood pressure monitor were applied, and a prophylactic antiemetic was administered (4 mg dexamethasone).

Induction of general anesthesia was achieved by 2 mg/kg propofol and 1 mcg/kg fentanyl; tracheal intubation was facilitated by 0.5 mg/kg atracurium after loss of consciousness. Anesthesia was maintained with isoflurane 1-1.2% in oxygen/air admixture and 0.1 mg/kg atracurium every 20 min. Additional analgesic boluses (1 mcg/kg fentanyl) were given when needed according to the attending anesthetist’s discretion.

Postoperatively, the VAS was assessed at rest (static) and during hip and knee flexion (dynamic; by asking the patient to bend her knee while in the supine position) at 0.5, 2, 4, 6, 10, 18, and 24 h after leaving the operating room. An intravenous morphine bolus (2 mg) was given when the VAS was > 3 (or at any time upon patient request) and can be repeated if the pain persisted for 30 min after the initial bolus. If postoperative nausea and vomiting occurred, intravenous 4-mg ondansetron was given.

The primary outcome was the mean postoperative dynamic VAS during the first 24 h. The secondary outcomes were static and dynamic VAS, time to first analgesic requirement (defined as the time from extubation until first analgesic requirement), intraoperative fentanyl requirements, postoperative morphine requirements, time to independent movement (defined as time from extubation until being able to move independently), intra- and postoperative hemodynamic measurements (heart rate and systolic blood pressure were recorded every 15 min intraoperatively, and at 0.5, 2, 4, 6, 10, 18 and 24 h postoperatively). The occurrence of opioid-related complications was recorded including nausea and vomiting (incidence and No. of episodes), itching, urine retention, sedation level using the Modified Ramsay Sedation Score, respiratory depression (defined as respiratory rate less than 8 breath per minute). At the end of the study, the participant was asked to evaluate her satisfaction with pain management on a scale of 0–10 (in which a score of 10 means strongly satisfied and a score of zero means strongly unsatisfied); We recorded the value at which the patient expressed her level of satisfaction. Patients’ demographic data (age, weight, body mass index, ASA), surgical characteristics (duration of surgery, need for blood transfusion, the use of vasopressors, amount blood loss [via visual assessment of surgical gauze and recording the amount of blood suctioned from the surgical field]), pre- and 24-h postoperative blood hemoglobin concentration were also recorded.

### Sample size

In a previous study, the difference in the mean dynamic VAS between the two groups was 1.45 with standard deviations of 2.29 and 2.43 for the ibuprofen and ketorolac groups, respectively [8]. We calculated the sample size using the mentioned standard deviations (2.29 and 2.43) and to detect a difference of 1.4 in the mean dynamic VAS. Having a study power of 80% and an alpha error of 0.05, the minimum number of patients would be 92. The number of envelopes was increased to 100 to compensate for possible dropouts. The sample size was calculated using the MedCalc (14.10.2) software.

### Statistical analysis

Statistical package for social science (SPSS) software, version 26 for Microsoft Windows (IBM. Corp., NY, USA) was used for data analysis. Categorical data are presented as frequency (%) and were analyzed by the Chi squared test or Fisher exact test as fitting. Continuous data were checked for normality using the Shapiro-Wilk test and are presented as mean ± standard deviation or median (quartiles) according to the data distribution. Unpaired continuous data were analyzed using the unpaired t test or Mann Whitney test according to the distribution of the data. Repeated measures were analyzed using the analysis of variance for repeated measures for normally distributed data (heart rate and systolic blood pressure). Area under the curve for the systolic blood pressure and heart rate readings over time was calculated and compared between the two groups. To compare the dynamic and static VAS values adjusted for the effect of time and morphine dose, we used a generalized estimating equation model that included the main effect of the group, time, and total morphine dose. The Bonferroni test was used to adjust for multiple testing. A *P*-value less than 0.05 was considered statistically significant.

## Results

**Fig. 1:**
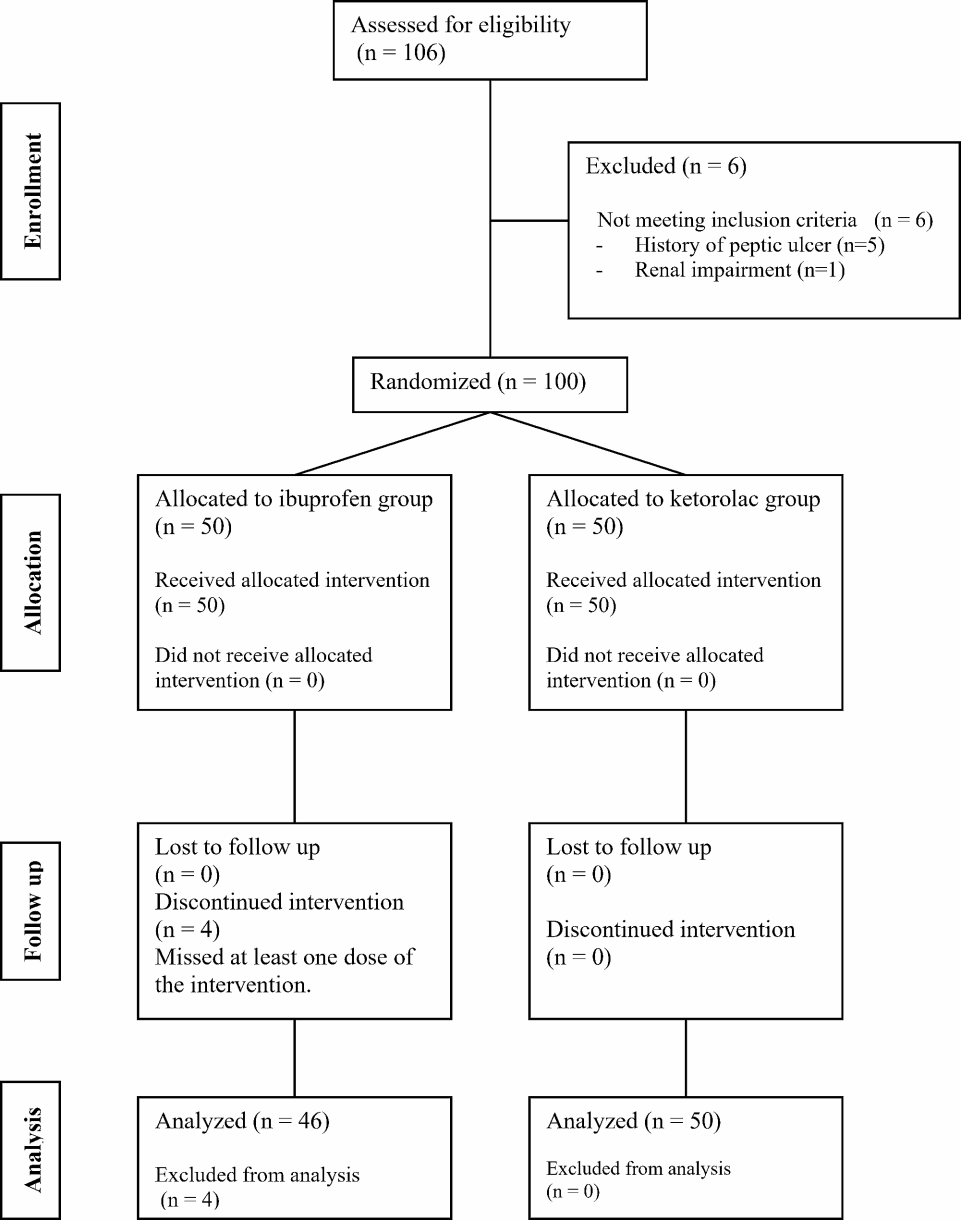
CONSORT’s flowchart

One-hundred and six patients were screened for eligibility, six patients were not included for not fulfilling the inclusion criteria, and 100 patients were randomized into one of the study groups. Four patients in the ibuprofen group were excluded from the analysis due to protocol violation in the form of not receiving one of the three doses. Therefore, 46 patients in the ibuprofen group and 50 patients in the ketorolac group were included in the final analysis. (Fig. 1)

The demographic data and surgical characteristics were similar between the two groups. (Table 1)

**Table 1:** Baseline data. Data are presented as mean ± standard deviation, median (quartiles) and frequency (%)

	Ibuprofen group (*n* = 46)	Ketorolac group (*n* = 50)
Age (years)	47 ± 7	48 ± 8
Weight (kg)	80 (70, 88)	83 (68, 89)
Height (cm)	165 (162, 167)	164 (160, 167)
Body mass index (kg.m^− 2^)	30 (27, 31)	30 (27, 32)
ASA-PS		
I	31 (70%)	37 (74%)
II	14 (30%)	13 (26%)
Comorbidity		
Hypertension Diabetes Mellites	0 (0%) 4 (9%)	2 (4%) 1 (2%)
Preoperative hemoglobin (gm/dL)	12.1 ± 1.3	11.8 ± 1.3
Incision type		
Pfannenstiel	45 (98%)	49 (98%)
Lower midline	1 (2%)	1 (2%)
Duration of the procedure (min)	75 (75, 109)	75 (60, 90)

ASA-PS: American society of anesthesiologist-physical status

**Fig. 2:**
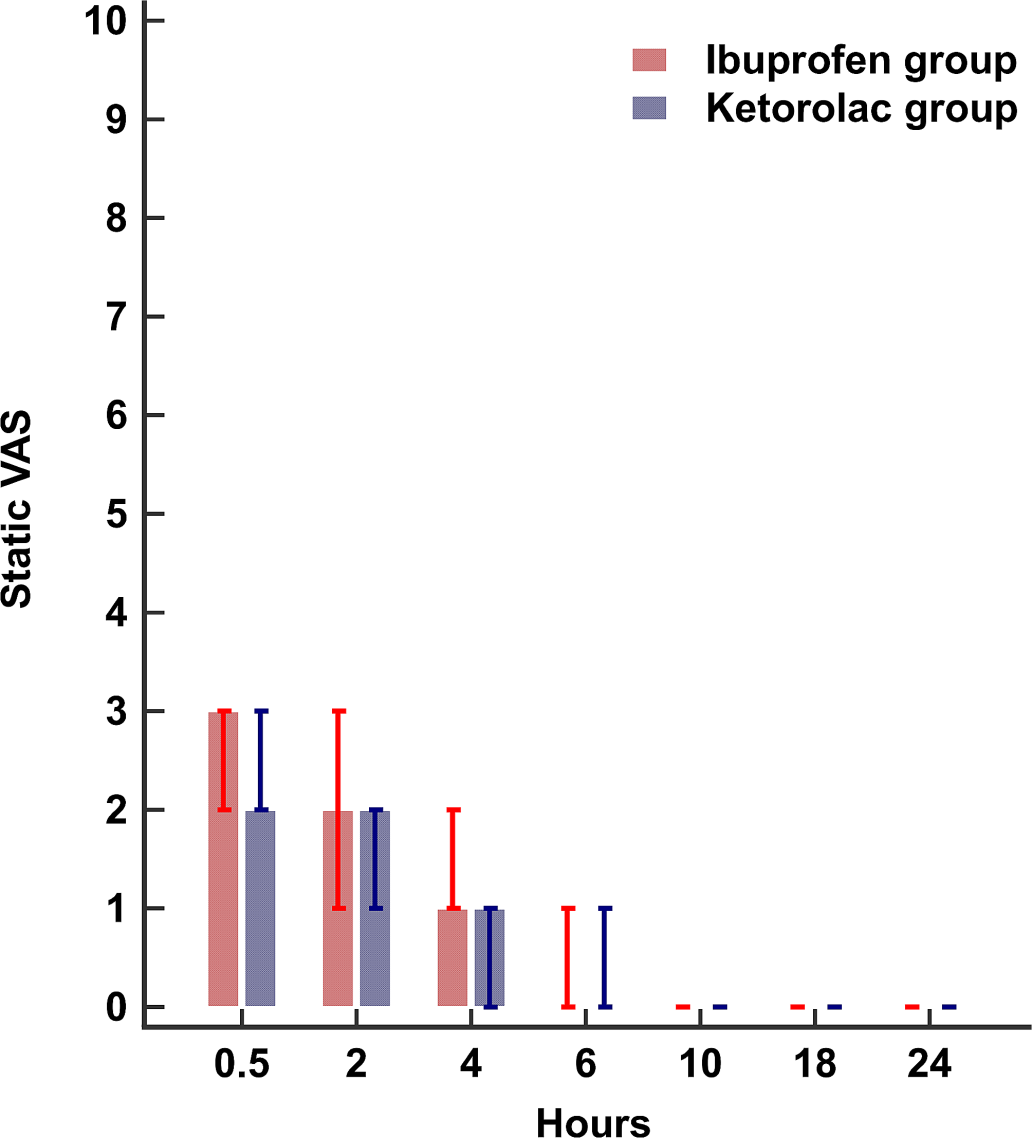
Bar and whisker blot for postoperative static VAS. Bars represent the median and whiskers represent the 25th and 75th percentiles. VAS: visual analogue scale

**Fig. 3:**
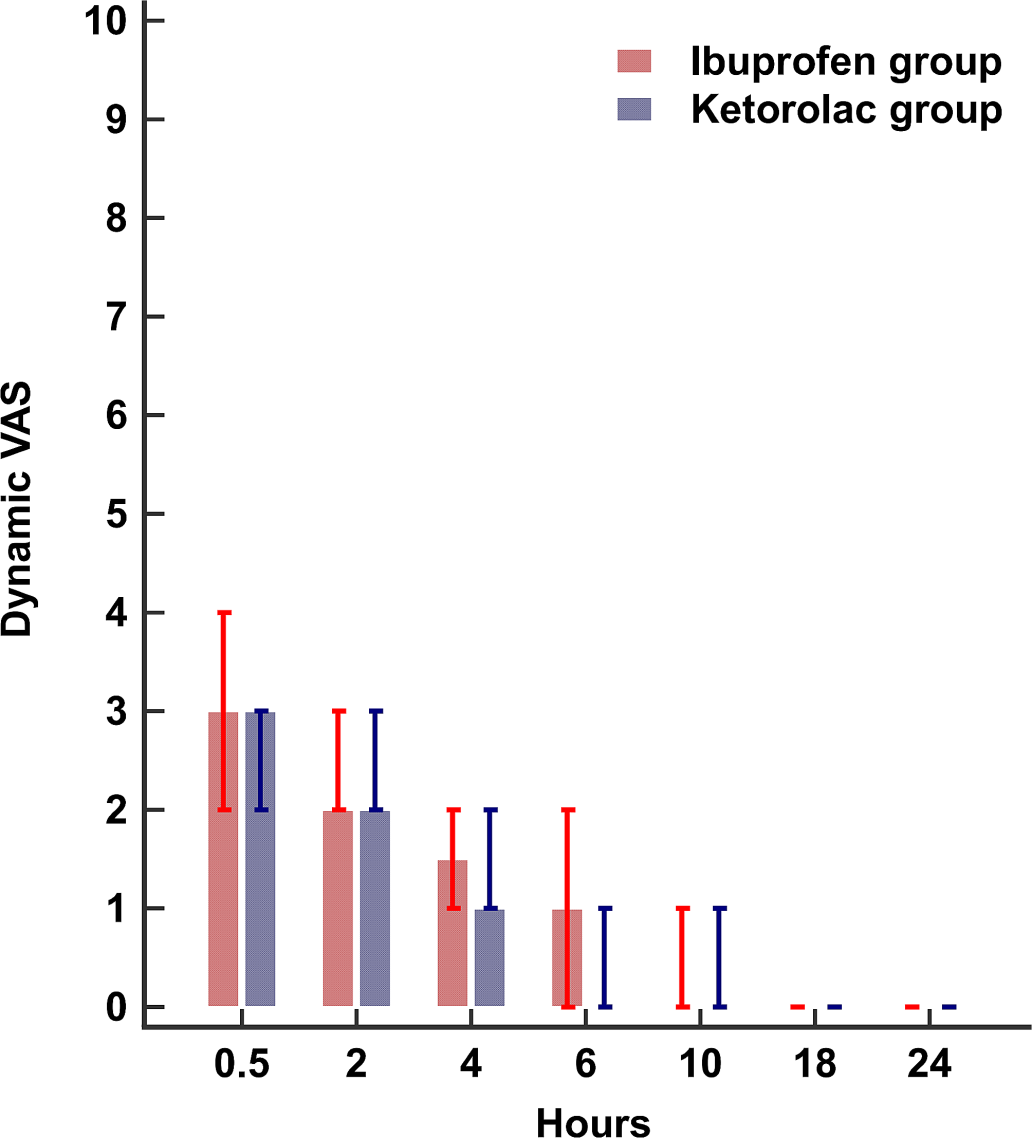
Bar and whisker blot for postoperative dynamic VAS. Bars represent the median and whiskers represent the 25th and 75th percentiles. VAS: visual analogue scale

The average 24-h static and dynamic VAS were similar between the two groups. (Table 2) (Figs. 2 and 3) (Supplementary Tables 1 and 2) Furthermore, after adjustment for the effect of time and morphine dose, the generalized estimating equation model for the VAS showed a between-group difference (95% confidence interval) of 0.06 (-0.07 to 0.17), *P*-value: 0.391, and 0.11 (-0.08 to 0.29) *P*-value: 0.269, for the static and the dynamic VAS, respectively.

**Table 2:** Perioperative analgesic requirement and postoperative VAS. Data are presented as median (quartiles), and frequency (%)

	Ibuprofen group (*n* = 46)	Ketorolac group (*n* = 50)	*P*-value
Intraoperative fentanyl requirement (mcg/kg)	2.1 (2.0, 2.9)	2.1 (2.0, 2.2)	0.519
Average 24-h static VAS	0.9 (0.6, 1.3)	0.7 (0.4, 1.1)	0.113
Average 24-h dynamic VAS	1.1 (0.9, 1.9)	1.0 (0.7, 1.3)	0.116
No. of patients needing morphine	46 (100%)	49 (98%)	1.000
Total morphine consumption (mg)	6 (4, 8)	6 (4, 7)	0.198
Time to independent movement (h)	4 (4, 4)	4 (4, 4)	0.621

VAS: visual analogue scale

The intra- and postoperative analgesic requirements were also similar between the two groups. (Table 2). All patients except one in the ketorolac group needed postoperative morphine (Table 2) and the time to first morphine requirement was within the first 30 min postoperatively.

The intraoperative blood loss, pre- and postoperative hemoglobin concentrations were similar between the two groups. The postoperative hemoglobin measurement decreased in comparison to the preoperative measurement within each group. (Table 3) None of the participants needed blood transfusion nor vasopressor intraoperatively.

**Table 3:** Other postoperative outcomes. Data are presented as mean ± standard deviation, median (quartiles), and frequency (%)

	Ibuprofen group (*n* = 46)	Ketorolac group (*n* = 50)	*P*-value
Intraoperative blood loss (mL)	455 (400, 600)	575 (400, 600)	0.319
Postoperative hemoglobin (gm/dL)	11.1 ± 1.3*	10.9 ± 1.3*	0.626
Postoperative Nausea and vomiting			
Incidence No. of episodes per patient Minimum-maximum No. of episodes	10 (22%) 0 (0, 0) 0–1	8 (16%) 0 (0, 0) 0–3	0.602 0.544
Postoperative modified RSS			0.536
Awake and alert	42 (91%)	43 (86%)	
Slightly sedated	4 (9%)	6 (12%)	
Moderately sedated	0 (0%)	1 (2%)	
Deeply sedated responds to nonpainful stimuli	0 (0%)	0 (0%)	
Deeply sedated responds to painful stimuli	0 (0%)	0 (0%)	
Deeply sedated unresponsive to painful stimuli	0 (0%)	0 (0%)	
Patient’s satisfaction	5 (4, 7)	6 (5, 7)	0.009

RSS: Ramsay sedation score. *Denotes significance in relation to the baseline hemoglobin within each group, P-value < 0.001 in both groups

The incidence of postoperative nausea and vomiting and level of sedation were not significantly different between the two groups. (Table 3) None of the study participants developed other opioid-related complications, namely itching, constipation, urine retention or respiratory depression. However, patient satisfaction was higher in the ketorolac group than the ibuprofen group.

The intra- and postoperative systolic blood pressure and heart rate generally decreased in relation to the baseline reading except for the early postoperative period where both increased in relation to the baseline reading. Both systolic blood pressure and heart rate readings were similar between the two groups. (Supplementary Fig. 1 and Supplementary Table 3) The area under the curve for the systolic blood pressure and heart rate were similar between the two groups with a P-value of 0.881 and 0.927, respectively.

## Discussion

This study compared intravenous ibuprofen and ketorolac in perioperative pain control in open hysterectomy surgery and its results revealed that the two drugs produced similar perioperative analgesic profile. All pain assessment parameters (pain scores, duration of analgesia, and consumption of analgesic drugs) were similar with administration of the two drugs; furthermore, ketorolac could be associated with higher patient satisfaction compared to ibuprofen.

Ketorolac had been the only approved intravenous NSAID for a long time until intravenous ibuprofen was approved in 2009 [7]. Both drugs exert their action through nonselective inhibition of COX enzyme which subsequently decreases prostaglandin production. The analgesic and anti-inflammatory effects are mediated by inhibition of COX-2 while the side effects are mediated by inhibition of COX-1 [9]. According to the Oxford League Table, the relative analgesic efficacy of ibuprofen and ketorolac was similar for acute pain management [9]. However, the data used to construct this table were pooled from heterogenous small studies and it is unclear which of the two drugs is more effective when used intravenously in different surgical settings. Therefore, their relative efficacy in pain prophylaxis in specific procedures is yet to be evaluated.

Ketorolac inhibits COX-1 and COX-2 enzymes with a ratio of 330:1 while ibuprofen inhibits the two enzymes with a ratio of 2.5:1 [10, 11]; therefore, it is hypothesized that ibuprofen could produce less side effects than ketorolac. Among the side effects of concern during the perioperative period is bleeding, due to platelet dysfunction [12]. A previous report had indicated that ketorolac increases the risk of postoperative surgical hematoma. Furthermore, platelet dysfunction following a single dose of ketorolac could be longer than that following ibuprofen administration [13]. In this study, the amount of blood loss was not significantly different between the two groups and none of the participant needed transfusion of blood products; however, the study was not powered to detect difference in this outcome and future studies are needed to confirm this observation.

Previous studies for the comparison of intravenous ketorolac and ibuprofen in abdominal surgery are sparse and no studies compared the two drugs in open laparotomy. Two previous randomized controlled trials compared the two drugs in laparoscopic procedures and their results were controversial. In line with our finding, Dwarica et al. showed no advantage for either drug in laparoscopic and robotic gynecological procedures [8]; however, subgroup analysis of the same study reported superiority of ketorolac over ibuprofen in patients who underwent laparotomy, representing 10% of the participants in the study [8]. The comparable results of the two drugs in our study differed from the subgroup of open laparotomy patients in Dwarica et al. study and this might be due to the larger number of patients in our study (100% our patients undergone laparotomy). Our patients received higher dose for acetaminophen (1 gm versus 650 gm in Dwarica et al.) and dexamethasone (which act as analgesic adjuvant) [14] and this might had minimized the effect of the study drugs and contributed in the comparable effects of both. This explanation is also supported by the lower resting and dynamic VAS in the two groups in our study (0.9 and 0.7) compared to Dwarica et al. study (4.9 and 2.8, respectively) [8]. Lee et al. reported that ketorolac was superior to ibuprofen in laparoscopic cholecystectomy [7]. Our study differed from Lee et al. results, probably for the impact of surgical site. Therefore, more research is warranted to specify the best drug for each operation in the presence of a multimodal protocol. We assume that among patients who receive several measures of analgesia, the difference between different molecules of NSAIDs would be minimal and the choice of the drug could be referred to the availability and cost.

Abdominal hysterectomy is a common operation and despite the increased trend towards the laparoscopic approach, open abdominal hysterectomy is still performed especially for large-sized uterus [15]. Therefore, evidence for perioperative analgesic plan for this procedure is needed. Proper analgesia would improve patient satisfaction, promote early ambulation, shorten the hospital stay and improve enhanced recovery programs. For the well-known short- and long term effects of opioid drugs, there is increased interest in opioid-sparing protocols in the postoperative period and several measures are used for this purpose [3]. Regional field blocks are effective routes of analgesia but they cannot stand alone as they do not cover visceral pain [16]. Neuraxial blocks produce dense analgesia at the expense of delayed ambulation and urine retention. Therefore, NSAIDs still represent a reputable and essential pillar for analgesia after surgery for being simple, effective; and devoid of upper airway and hemodynamic complications [17], when not contraindicated [18, 19]. Furthermore, NSAIDs allow effective control of dynamic pain which promote early ambulation [20] resulting in their inclusion in analgesia protocol in various procedure specific guidelines [14, 21–23]. According to our results, we support the use of ketorolac over ibuprofen because it produced similar analgesia, with the advantage of being more economic. Furthermore, patients’ satisfaction was slightly higher in the ketorolac group than the ibuprofen group; however, the current study was not powered for this outcome and future studies are needed to confirm this finding.

In this study, we used ibuprofen at a dose of 800 mg since it is the most used in previous reports [24]; furthermore, it is the maximum allowed single dose for the drug. Since there are no data regarding the equipotent dosing of intravenous formulation of NSAIDs; we also used the maximum allowed dose for ketorolac [25].

This study had several strengths such as being a randomized controlled double-blinded design. Furthermore, the 95% confidence interval of the VAS difference between the two drugs was narrow (-0.07 to 0.17 point for static VAS and − 0.08 to 0.29 point for dynamic VAS); which confirms that a clinically significant difference between the two drugs is less likely in our patients.

There are some limitations such as using one dose of each drug, the lack of local anesthetic wound infiltration, and the limited duration of follow-up. In this study, we used dexamethasone in a dose of 4 mg for prophylaxis against postoperative nausea and vomiting and as an analgesic adjuvant [26]. Since the analgesic effect of dexamethasone is dose-dependent [14], future studies are needed to explore the efficacy of the two drugs in the presence of higher dose of dexamethasone (> 4 mg). In this study, we assessed the dynamic VAS through hip and knee flexion during the supine position; Currently there is no standardized technique for movement-evoked pain [27], and we believe that the chosen maneuver is appropriate for this type of procedure. However, future studies are needed to evaluate the dynamic VAS using other maneuver such as cough. Another limitation in this study is the absence of intension-to-treat analysis. Patients with incomplete intervention (not receiving one of the three scheduled doses) were excluded from the analysis since their data were not complete and their collection data sheets were not available at the time of the analysis.

In conclusion, the two study drugs, intravenous ibuprofen and ketorolac produced similar analgesic profile in patients undergoing open abdominal hysterectomy receiving multimodal analgesic regimen.

### Electronic supplementary material

Below is the link to the electronic supplementary material.

Supplementary Material 1

Supplementary Material 2

Supplementary Material 3

Supplementary Material 4

## Data Availability

The datasets used and/or analyzed during the current study are available from the corresponding author on reasonable request.
