# Supplementary material for: Intravenous ibuprofen versus ketorolac for perioperative pain control in open abdominal hysterectomy: a randomized controlled trial
Source: BMC Anesthesiol. 2024 Jun 7;24:202. doi: 10.1186/s12871-024-02571-0 (PMC11157756; doi:10.1186/s12871-024-02571-0)
Supplement: Supplementary file 2 — Supplementary Material 2 [file 12871_2024_2571_MOESM2_ESM.docx]

**Intravenous ibuprofen versus ketorolac for perioperative pain control in open abdominal hysterectomy: a randomized controlled trial**

Sarah Amin (MD)^1^, Ahmed Hasanin (MD, DESA)^1^, Ola A Attia (MSc)^1^, Maha Mostafa (MD)^1^, Nashwa S Elzayat (MD)^1^, Mona Elsherbiny (MD)^1^, Amany A Eissa (MD)^1^

^1^ Department of Anesthesia and Critical Care Medicine, Cairo University, Cairo, Egypt

Supplementary Table 2: Dynamic VAS. Data are presented as count and percentage.

|  |  | Ibuprofen group (n=46) | | ketorolac group (n=50) | |
| --- | --- | --- | --- | --- | --- |
| Time | Dynamic VAS | Count | % | Count | % |
| 0.5 h | 1 | 0 | 0% | 4 | 8% |
|  | 2 | 12 | 26% | 13 | 26% |
|  | 3 | 17 | 37% | 22 | 44% |
|  | 4 | 10 | 22% | 6 | 12% |
|  | 5 | 4 | 9% | 4 | 8% |
| 2 h | 0 | 2 | 4% | 1 | 2% |
|  | 1 | 9 | 20% | 10 | 20% |
|  | 2 | 13 | 28% | 22 | 44% |
|  | 3 | 13 | 28% | 14 | 28% |
|  | 4 | 7 | 15% | 2 | 4% |
| 4 h | 0 | 8 | 17% | 11 | 22% |
|  | 1 | 15 | 33% | 23 | 46% |
|  | 2 | 13 | 28% | 7 | 14% |
|  | 3 | 9 | 20% | 9 | 18% |
|  | 4 | 1 | 2% | 0 | 0% |
| 6 h | 0 | 17 | 37% | 26 | 52% |
|  | 1 | 17 | 37% | 17 | 34% |
|  | 2 | 10 | 22% | 6 | 12% |
|  | 3 | 2 | 4% | 1 | 2% |
| 10 h | 0 | 29 | 63% | 35 | 70% |
|  | 1 | 14 | 30% | 14 | 28% |
|  | 2 | 3 | 7% | 1 | 2% |
|  | 0 | 35 | 76% | 42 | 84% |
| 18 h | 1 | 10 | 22% | 7 | 14% |
|  | 2 | 0 | 0% | 1 | 2% |
|  | 3 | 1 | 2% | 0 | 0% |
| 24 h | 0 | 37 | 80% | 42 | 84% |
|  | 1 | 8 | 17% | 7 | 14% |
|  | 2 | 1 | 2% | 1 | 2% |

VAS: visual analogue scale
