# Supplementary material for: Intravenous ibuprofen versus ketorolac for perioperative pain control in open abdominal hysterectomy: a randomized controlled trial
Source: BMC Anesthesiol. 2024 Jun 7;24:202. doi: 10.1186/s12871-024-02571-0 (PMC11157756; doi:10.1186/s12871-024-02571-0)
Supplement: Supplementary file 3 — Supplementary Material 3 [file 12871_2024_2571_MOESM3_ESM.docx]

**Intravenous ibuprofen versus ketorolac for perioperative pain control in open abdominal hysterectomy: a randomized controlled trial**

Sarah Amin (MD)^1^, Ahmed Hasanin (MD, DESA)^1^, Ola A Attia (MSc)^1^, Maha Mostafa (MD)^1^, Nashwa S Elzayat (MD)^1^, Mona Elsherbiny (MD)^1^, Amany A Eissa (MD)^1^

^1^ Department of Anesthesia and Critical Care Medicine, Cairo University, Cairo, Egypt

Supplementary table 3: Details of systolic blood pressure and heart rate data

| **Systolic blood pressure(mmHg)** | | | | | | | | | | | |
| --- | --- | --- | --- | --- | --- | --- | --- | --- | --- | --- | --- |
|  |  | Intraoperative | | | | Postoperative | | | | | |
|  | 0 | 0.25 | 0.5 | 0.75 | 1 | 2 | 4 | 6 | 10 | 18 | 24 |
| Ibuprofen group(n=46) | | | | | | | | | | | |
| Mean | 132 | 123 | 125 | 125 | 122 | 132 | 127 | 126 | 123 | 122 | 121 |
| 95% CI for Mean | 127-136 | 116-130 | 119-131 | 120-129 | 118-127 | 128-137 | 124-130 | 122-129 | 120-126 | 119-124 | 118-124 |
| SD | 16 | 23 | 19 | 16 | 14 | 15 | 11 | 13 | 10 | 8 | 11 |
| Median | 134 | 121 | 125 | 128 | 121 | 133 | 129 | 127 | 125 | 121 | 120 |
| 25^th^ percentile | 119 | 101 | 111 | 112 | 112 | 125 | 120 | 120 | 120 | 120 | 115 |
| 75^th^ percentile | 143 | 136 | 137 | 137 | 132 | 143 | 134 | 135 | 130 | 129 | 130 |
| Ketorolac group (n=50) | | | | | | | | | | | |
| Mean | 131 | 121 | 120 | 118 | 118 | 129 | 124 | 122 | 121 | 119 | 118 |
| 95% CI for Mean | 126-136 | 117-126 | 115-125 | 114-122 | 114-122 | 126-133 | 121-128 | 118-125 | 117-124 | 115-122 | 115-120 |
| SD | 17 | 17 | 16 | 14 | 14 | 12 | 12 | 12 | 12 | 13 | 9 |
| Median | 132 | 120 | 118 | 119 | 117 | 131 | 125 | 120 | 120 | 120 | 120 |
| 25^th^ percentile | 120 | 111 | 111 | 110 | 108 | 120 | 115 | 114 | 110 | 110 | 110 |
| 75^th^ percentile | 142 | 130 | 130 | 127 | 127 | 136 | 130 | 130 | 130 | 130 | 123 |
|  |  |  |  |  |  |  |  |  |  |  |  |
| **Heart rate (bpm)** | | | | | | | | | | | |
|  |  | Intraoperative | | | | Postoperative | | | | | |
|  | 0 | 0.25 | 0.5 | 0.75 | 1 | 2 | 4 | 6 | 10 | 18 | 24 |
| Ibuprofen group (n=46) | | | | | | | | | | | |
| Mean | 87 | 82 | 78 | 78 | 75 | 82 | 78 | 73 | 73 | 72 | 73 |
| 95% CI for Mean | 84-90 | 78-86 | 74-82 | 74-81 | 72-78 | 80-84 | 75-80 | 71-75 | 71-75 | 70-74 | 71-75 |
| SD | 11 | 14 | 13 | 11 | 10 | 7 | 7 | 7 | 6 | 7 | 6 |
| Median | 87 | 80 | 79 | 77 | 74 | 81 | 76 | 72 | 73 | 72 | 73 |
| 25^th^ percentile | 80 | 72 | 70 | 70 | 68 | 77 | 72 | 68 | 68 | 68 | 70 |
| 75^th^ percentile | 93 | 92 | 85 | 84 | 81 | 87 | 83 | 77 | 80 | 76 | 76 |
| Ketorolac group(n=50) | | | | | | | | | | | |
| Mean | 84 | 82 | 77 | 75 | 73 | 80 | 75 | 73 | 72 | 71 | 71 |
| 95% CI for Mean | 81-87 | 78-85 | 73-80 | 72-79 | 70-77 | 77-82 | 72-77 | 71-75 | 70-74 | 69-72 | 69-73 |
| SD | 11 | 13 | 13 | 12 | 11 | 9 | 9 | 8 | 7 | 6 | 7 |
| Median | 87 | 84 | 78 | 74 | 73 | 80 | 76 | 74 | 72 | 70 | 72 |
| 25^th^ percentile | 77 | 72 | 65 | 67 | 67 | 75 | 65 | 67 | 67 | 67 | 65 |
| 75^th^ percentile | 92 | 90 | 85 | 82 | 78 | 86 | 82 | 80 | 77 | 75 | 75 |

CI: confidence interval, SD: standard deviation
