# Supplementary material for: Intravenous ibuprofen versus ketorolac for perioperative pain control in open abdominal hysterectomy: a randomized controlled trial
Source: BMC Anesthesiol. 2024 Jun 7;24:202. doi: 10.1186/s12871-024-02571-0 (PMC11157756; doi:10.1186/s12871-024-02571-0)
Supplement: Supplementary file 4 — Supplementary Material 4 [file 12871_2024_2571_MOESM4_ESM.docx]

**Intravenous ibuprofen versus ketorolac for perioperative pain control in open abdominal hysterectomy: a randomized controlled trial**

Sarah Amin (MD)^1^, Ahmed Hasanin (MD, DESA)^1^, Ola A Attia (MSc)^1^, Maha Mostafa (MD)^1^, Nashwa S Elzayat (MD)^1^, Mona Elsherbiny (MD)^1^, Amany A Eissa (MD)^1^

^1^ Department of Anesthesia and Critical Care Medicine, Cairo University, Cairo, Egypt


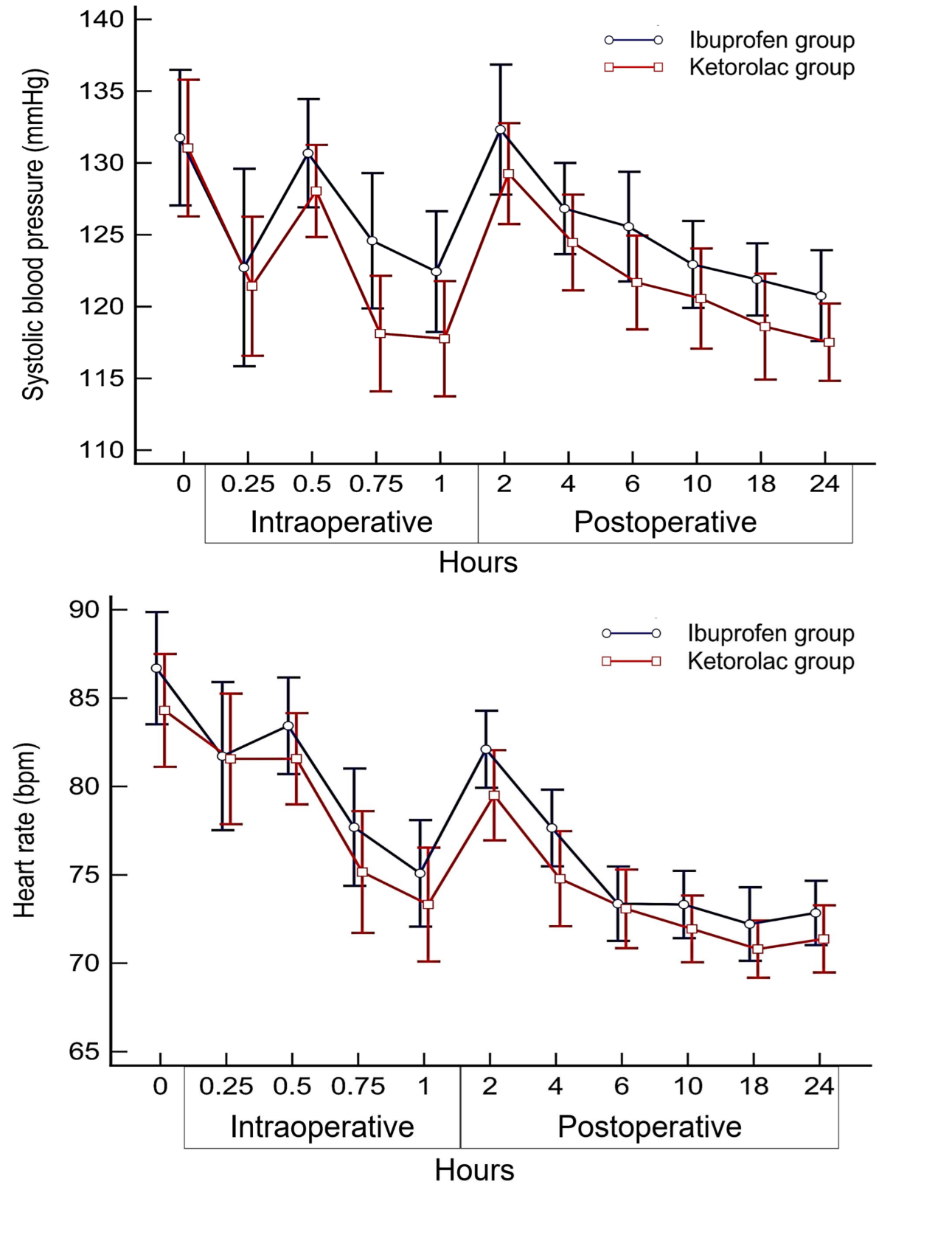


Supplementary figure 1: Systolic blood pressure (above) and Heart rate (below). Markers are the mean and error bars are the 95% confidence interval of the mean.
